# Chicago Medical Society

**Published:** 1883-05

**Authors:** 


### Society Reports.

---

ARTICLE VI.

CHICAGO MEDICAL SOCIETY.

The society held its semi-monthly meeting March 19, 1883, with a very large attendance, Dr. J. H. Hollister, President, in the chair.

Drs. F. S. Tabor; A. R. Reynolds; T. A. Stanley; E. W. Andrews; A. Goldspoon; J. E. Best and F. E. Wadhams were duly elected to full membership.

Dr. N. S. Davis read the paper for the evening, on the subject of acute pneumonia, or lung fever.

During 1882, 782 deaths occurred in Chicago from pneumonia; 42 from broncho-pneumonia; 20 from pleuro-pneumonia; and of this number (844) 122 died in January, 1882. The census reports show that in Boston 681 died from pneumonia in 1882, and that in San Francisco 527 died from pneumonia in the same year. The deaths from pneumonia in New Orleans in 1882 were 203. In San Francisco, the Mongolian race died in the proportion of two to one of the Caucasian race, and in New Orleans, three colored persons to one white died of the disease--accounted for, in a great measure, by unsanitary conditions of the Chinese and colored races. Another destroying agent was the evil effects of alcoholic drinks that those races were addicted to, which increased the death rate.

Dr. Davis then recited a typical case of pneumonia, which resulted in the death of a girl æt. fourteen, giving the minute symptoms in detail, which were interesting and curious, as the brain and pleuritic complications were present, and rapidly resulted in death in five days. Another curious case, with prominent brain symptoms, occurred in a girl older than the case just cited. In this case, endo-carditis set in, and she died in five days, with general convulsions. In these two cases, he questioned whether the pneumonia was not the result of the cerebral symptoms, that occurred first by a partial paralyzing of the phrenic nerve, to bring on these sudden changes.

*Sources of danger in pneumonia.*—The first is in the stage of congestion. This may gradually smother the patient. He cited two cases of this, but it rarely results fatally. A second danger is from hepatization of the lung, which is the result of depletion of blood, and its being solidified in the lung; it is depleted from the circulation. A third critical period is when suppurative degeneration has supervened—this gradually, day by day, exhausts patients. The fourth danger is from rapid reflex changes which may occur, such as "spotted fever." These erratic symptoms may appear almost epidemically at certain periods or places. Of the remedies, venesection is really the best, and will ease patients if done just before the stage of engorgement or solidification sets in; then follow this with proper remedies to keep the patient relieved, and to favor resolution.

If hepatization has set in before we have seen the patient, do not give alcoholic stimulants, but give good strong tea and coffee, (or theine and caffeine) also give chlorate of potash in gum arabic small solution, and doses of quinine, strychnine, camphor or carbonate of ammonia, and the mineral acids.

In the suppurative stage, we should keep up nutrition as best we can, by supporting the patient's strength with tonics and good nourishment.

DISCUSSION.

Dr. R. C. Hamill said that far more pneumonia prevailed in the hilly counties of Indiana than in this city. The lancet was the remedy used fifty years ago, and it was successful then. Within a few days he had had a singular case, which apparently was much improved, and yet died in fifteen minutes after the doctor left. The man died, he thought, from embolism as a complication of the pneumonia.

Dr. G. H. Randall asked Dr. Davis if it was not possible that quinine or opium might cut short the congestive stage of pneumonia, the same as blood-letting would?

Dr. H. A. Johnson thought it probable that the death rate varied in different years; the statistics varied in this. Regarding the peculiar phases of the disease, he could hardly add anything to that which had already been stated in the paper. When hepatization occurs, the process occupies days, and the abstraction, therefore, is gradual, and two or three lbs. at most from hepatization, equal to 16 oz. of blood suddenly taken by bleeding a patient. The continued doses of alcohol in large quantities he thought seemingly resulted favorably in these cases of cardiac asthenia, or in the extreme prostration of pneumonia. He endorsed venesection in the early stages of the disease.

Dr. C. T. Fenn recited a case of a boy, æt. fourteen, who died of pneumonia, who had no symptoms of the disease except the sputum that was expectorated. He died on the twenty-second day of his sickness, while taking a dose of medicine, and he died suddenly.

Dr. A. J. Avery had used carbonate of ammonia with a great degree of satisfaction, in cases where the patients had appeared almost at death's door.

Dr. J. H. Hollister thought that in malarial districts, if a person took pneumonia, the disease is more fatal in a given number of cases, than in healthy districts.

Dr. Davis answered Dr. Randall's question, by saying he had tried giving quinine and opium at the onset of the disease; it was well to do so in some cases, but he mentioned two cases where he had first given these remedies, and afterward had to bleed the patients before relief was afforded.

REPORT OF THE COMMITTEE ON BANQUET.

Dr. D. R. Brower, of the committee, wished instructions from the society as to how much money should be expended?

Dr. Davis wanted to know if they were to come together to feast simply, as he did not favor wine.

A vote, taken to ascertain those in favor of a banquet, to be paid for from the society's fund, was lost.

Another vote, taken of those in favor of a banquet, providing 100 were guaranteed, and $2.00 a plate charged, each paying from his own pocket, was carried.

Dr. C. T. Fenn wished to know the object of the banquet. Was it to become acquainted with each other, and have a social time? if so, he was in favor of it; he himself was not acquainted with fifteen members of the society.

Dr. Avery asked if members could invite their wives to the banquet, and was answered that it would be proper to bring them, as this was done in the National Medical Association banquets. It was upon vote decided that the wives or any friend of the members might be invited, and ladies would be cordially welcome to this banquet, the first ever having been held by the society.

The following members signified their intention of attending the banquet to be held here, at the Grand Pacific Hotel, at the annual meeting, April 2:

A. H. Tagert; E. Bert; W. Blanchard; C. T. Fenn; H. A. Johnson; J. H. Chew; S. J. Avery; R. C. Hamill; E. G. R. Trimble; L. T. Potter; A. H. Cooke and wife; J. F. Todd; N. S. Davis and wife; D. M. Tucker; R. L. Leonard and one other; J. H. Bates and two others; E. Ingals; W. H. Curtis; J. H. Hollister and wife; L. H. Montgomery and wife; J. E. Walton; A. J. Coey; L. J. Loescher.

A communication from the corresponding secretary of the Illinois Microscopical Society to this society was read, inviting this society to join with them in tendering to the American Society of Microscopists a welcome when they hold their annual meeting in this city next August, and for the President to appoint a committee of one or more to act with their committee of arrangements.

Dr. E. Ingals moved that the communication be received and adopted, and that the President appoint the committee when convenient for him to do so.

Dr. W. H Curtis moved that a committee of five on nomination be appointed to nominate officers for the coming year.

Dr. S. J. Avery moved that the meeting be at 7 o'clock (annual meeting); seconded by Dr. Davis. The business meeting for April 2 will be held at 7 P. M.

The committee of five on nominations consisted of Drs. J. H. Hollister; E. Ingals; N. S. Davis; W. H. Curtis; H. A. Johnson.

The society then adjourned.

L. H. MONTGOMERY, *Secretary.*

---

The Chicago Medical Society held the Thirty-third Annual Meeting, April 2, 1883, with Dr. J. H. Hollister in the President's chair and Dr. L. H. Montgomery at the Secretary's desk. There was a very large percentage of members present.

Dr. F. E. Waxham read a paper on the "Prevention of Contagious Diseases" and "Our Hospital Accommodations for Children." He held that there is a wide field for earnest labor in the prevention of contagious diseases; children of all classes, conditions and localities are subject to them. The unnecessary and avoidable waste of life due to this cause is great indeed, and, by the systematic use of disinfectants, antiseptics and isolation, we certainly possess the means of controlling these maladies, the same as by vaccination in preventing small-pox; although for scarlet fever and other contagious diseases we have, as yet, no specific preventive; but by the above means and carbolized oil, or carbolized vaseline, applied direct to their little bodies, we ought to control these diseases, and much can be gained by the use of a five per cent. solution of carbolic acid with the hand or steam atomizer; and, to be effectual, it should be used as often as every two or three hours in the room and about the bed of the patient. Measles and scarlet fever can thus be prevented from spreading. and for whooping-cough isolation is the most important measure, Cases and illustrations under the writer's care were given to substantiate these facts. Among the poorer class of tenants, who are overcrowded, contagious diseases occur with the greatest frequency, and are most severe and fatal. It is for this class especially in behalf of which the paper was written, and two methods are practicable to protect them, viz.: Either by the erection of hospitals for the sick, or asylums for the well, as the exposure incident to the removal of a child suffering from scarlet fever, measles, or diphtheria may be such as to induce a fatal result. If a hospital or asylum were erected here exclusively for children, hundreds of lives might be saved annually. In this city, which has become a great medical center, with our magnificent hospitals, the little children have been overlooked. This is not so in New York and some other of the Eastern cities. Forty-five thousand children are treated annually in the hospitals of New York City. The Thomas Wilson Sanitarium for children has been founded at a cost of $500,000 in Baltimore, and with all the wealth and generosity of Chicago, but a single hospital exclusively for children may be found—the Maurice Porter Hospital of the North Side, containing twelve beds. The Home for the Friendless and Foundlings' Home have ward accommodations simply for the inmates, and are not accessible to outside patients. How different, if there existed here a complete and exclusive hospital for children, with wards for the different diseases of which they may become afflicted! Besides, it would be a refuge for the unexposed. The little hospital of the North Side mentioned in the paper is inadequate to the demands and needs of this great city. Here is an opportunity for the philanthropist to make his name immortal, or for the Chicago Medical Society's influence—inducing legislation, donations and bequests. It is the proper body to interest itself, and soon we would see hospital accommodations for the little children worthy the enterprise, wealth and generosity of Chicago.

The author moved, therefore, that a committee of three be appointed to agitate this subject. Carried; to be appointed at a future meeting.

The annual report of the Secretary was read, which, in brief, summarized, is as follows: During the year twenty meetings for scientific purposes have been held; 450 members attended nineteen of the meetings and 120 visitors, being a total of 570, or an average of thirty persons. The Society admitted thirty-four physicians into full membership; the accurate and corrected membership list now is 210. Postal card announcements sent out for each meeting were 200, or about 4,200 for the entire year. The list of papers presented during the year with authors was thirty-eight, many of them being of unusual interest learned and comprehensive. No deaths occurred and four marriages, which is probably the best part of this report.

The report closed with the following suggestions for future consideration:

*First.* That regarding the amendment to the constitution proposed at the second preceding meeting (by myself), wherein provision for a second Vice-president should be made, for the reason set forth at the time it was offered.

*Second.* That no member be permitted to present more than two new candidates during a year.

*Third.* That a Publication Committee, consisting of three members, be appointed by the President, and a given number of copies of our constitution and by-laws be published in pamphlet form, with the list of membership and other information contained, to supply each member with a copy.

*Fourth.* That a "Professional Insurance" be established for beneficiary purposes out of the funds in the treasury. We then have the double advantage and honor of belonging to a medical society the peer of any west of New York, as well as this relief fund, at a mere nominal outlay for a member's death.

In closing, the report stated that never had the Society been in so prosperous a condition, and for this result much honor was due the worthy President.

Dr. E. Ingals moved that the Secretary's report be received and adopted. Carried unanimously. And that a committee of three be appointed to consider it. Also carried, resulting in Dr. E. Ingals, Dr. D. W. Graham and Dr. R. G. Bogue constituting the committee.

The Treasurer, Dr. E. F. Ingals, presented his report for the fiscal year to date:

| | | |
|---|---|---|
| Dr. | | |
| April 13, 1882, to amount in Treasury, | $339 07 | |
| " 13 to July 1, 1882, Collections, | 25 00 | |
| July 1st to January 2, 1883, Collections | 83 00 | |
| January 2, 1883 to April 2, 1883, Collections, | 230 00 | |
| Cr. | $677 07 | |
| April 13th to July 1, 1882, by bills paid, | | $ 68 50 |
| July 1st to January 2, 1883, by bills paid, | | 24 25 |
| January 2d to April 2, 1883, by bills paid, | | 36 50 |
| | | $129 25 |

Leaving a balance of $547.82 in the treasury.

Dr. E. Ingals moved that the treasurer's report be received, and that an auditing committee be appointed consisting of two. Carried, resulting in Dr. C. T. Fenn and the Secretary elect being appointed.

The committee on nominations reported for the current year, as follows: For President, Dr. D. W. Graham; Vice-President, Dr. R. N. Isham; Secretary, Dr. L. H. Montgomery; Treasurer, Dr. E. F. Ingals.

Dr. T. W. Miller thought other nominations should be made, as we probably would have a second Vice-President. It was so ordered, and upon balloting a number of times, the following officers were elected for the coming year: Dr. D. W. Graham, President; Dr. R. G. Bogue, first Vice-President; Dr. R. Park, second Vice-President; Dr. L. H. Montgomery, Secretary; Dr. E. F. Ingals, Treasurer. Doctors G. C. Paoli, C. T. Fenn and D. R. Brower to constitute the Committee on Membership and miscellaneous business.

Dr. Hollister, the retiring President, made some happy remarks, and dwelt upon the future for us. He appointed Dr. E. F. Ingals to escort the newly elected President to the chair. Dr. Graham, upon accepting the Presidency, thanked those present for this honor.

Dr. Paoli moved that the retiring President receive a vote of thanks for the impartial manner and efficient services rendered. Unanimously carried.

Dr. E. Ingals moved that $50.00 be presented to Mr. Drake for courtesy in using the parlors of the Grand Pacific hotel. Carried unanimously.

Dr. E. Ingals also moved that the Secretary, Dr. L. H. Montgomery be presented, as an honorarium or testimonial for services, the sum of $25.00. Carried unanimously.

The society then adjourned. L. H. M.

At a called meeting of the Ogle County Medical Society, held in the city of Oregon, March 7th, 1883, the following preamble and resolutions were unanimously adopted:

WHEREAS, The Ogle County Medical Society is again called upon to register the loss of one of its oldest and most honored members, by the death of Dr. Elias S. Potter; the physicians of Ogle county lose a worthy colleague who has labored and dwelt among us for more than forty years. He made his home in the city of Oregon in 1844, and acquired a very large practice, enjoying the love and confidence of his patients and friends as a most estimable man. In the midst of his professional duties, and in the very act of visiting the sick, he was called suddenly away. The Ogle County Medical Society give voice to the unanimous sentiment of the profession in the expression of their sorrow at the sudden demise of Dr. Potter and their deep sympathy with the bereaved family; therefore,

*Resolved*, That in the death of Dr. Potter the medical profession of Ogle County has lost one of its most honorable, upright and most respected brothers, whose judgment, kindness and genuine congeniality we have always esteemed and admired.

*Resolved*, That we sincerely mourn and deplore the loss of our brother whose gentlemanly conduct, generosity of heart, and professional ability were the characteristics of his life.

*Resolved*, That in the labor and character of our brother, we have an example of industry, manliness and usefulness, in a high degree worthy and commendable our imitation.

*Resolved*, That we tender the bereaved family of the deceased our heart-felt sympathy and condolence in this, their sad bereavement and affliction.

*Resolved*, That a copy of these resolutions of respect and condolence be sent to the family, also to the Ogle County papers for publication, and to the *Chicago Medical Journal and Examiner*.

DRS. H. A. MIX, L. C. HORMELL,<br>L. S. HALL, W. T. SPEAKER, } Committee.

